# *Lactiplantibacillus plantarum* CQPC03 Reduces Carrageenan‐Induced Thrombosis in Mice via Controlling the Flora in the Gut

**DOI:** 10.1002/fsn3.71842

**Published:** 2026-05-01

**Authors:** Xin Zhao, Kejia Wang, Tianyi Chen, Kai Ma, Rongming Zhang, Yongling Ou, Zhiping Kuang

**Affiliations:** ^1^ Collaborative Innovation Center for Child Nutrition and Health Development, Chongqing Engineering Research Center of Functional Food, Chongqing Engineering Laboratory for Research and Development of Functional Food Chongqing University of Education Chongqing People's Republic of China; ^2^ Department of Geriatric Medicine Chongqing Seventh People's Hospital Chongqing People's Republic of China; ^3^ Shandong Port Land‐Sea (Jinan) Hotel Management Co. Ltd. Jinan Shandong People's Republic of China; ^4^ Jiangsu New‐Bio Biotechnology Co. Ltd. Wuxi Jiangsu People's Republic of China; ^5^ Centre Testing International (Qingdao) Co. Ltd. Qingdao Shandong People's Republic of China; ^6^ The First Department of Orthopaedic Surgery Chongqing Traditional Chinese Medicine Hospital Chongqing People's Republic of China

**Keywords:** anti‐inflammation, carrageenan, *Lactiplantibacillus plantarum*, oxidative stress, thrombosis

## Abstract

This investigation reports the effects of *Lactiplantibacillus plantarum* CQPC03 (LP‐CQPC03) on preventing thrombus formation and reducing intestinal oxidative stress and inflammation in a carrageenan‐induced mouse thrombosis model. High‐throughput 16S rRNA sequencing determined the composition of intestinal microorganisms. Biochemical reagents, section observations, and quantitative polymerase chain reaction (qPCR) identified mouse serum and tissue‐related markers. Experimental findings show that LP‐CQPC03 enhances activated partial thromboplastin time (APTT) and decreases thrombin time (TT), fibrinogen (FIB), and prothrombin time (PT). LP‐CQPC03 also significantly reduces black tail severity in thrombotic mice. Moreover, LP‐CQPC03 lowers malondialdehyde (MDA), tumor necrosis factor alpha (TNF‐α), interleukin‐6 (IL‐6), nuclear factor kappa‐B (NF‐κB), and interleukin‐1 beta (IL‐1β) levels in thrombotic mouse serum, while increasing superoxide dismutase (SOD) and catalase (CAT) activities. Hematoxylin and eosin (H&E) pathological analysis reveals that LP‐CQPC03 lessens tissue damage caused by tail vein thrombosis. In the colon tissues of thrombotic mice, LP‐CQPC03 up‐regulates the mRNA expression of copper/zinc superoxide dismutase (Cu/Zn‐SOD), manganese superoxide dismutase (Mn‐SOD), and CAT, while down‐regulating NF‐κB p65, IL‐6, TNF‐α, and interferon gamma (IFN‐γ). In tail vein vascular tissues, LP‐CQPC03 also suppresses the mRNA expression of NF‐κB p65, intercellular cell adhesion molecule‐1 (ICAM‐1), vascular cell adhesion molecule‐1 (VCAM‐1), and E‐selectin. Gut microbiota sequencing results show that LP‐CQPC03 increases the population of beneficial bacteria and decreases harmful ones. These findings demonstrate that LP‐CQPC03 prevents thrombosis in mice, reduces oxidative stress and intestinal inflammation, and regulates gut microbiota by increasing beneficial bacteria. A high concentration of LP‐CQPC03 shows a better effect, comparable to heparin.

## Graphical abstract

*Lactiplantibacillus plantarum* CQPC03 could reduce carrageenan‐induced thrombosis in mice.

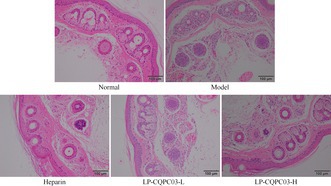

## 1 Introduction

In recent years, thrombosis has become a high‐incidence disease in middle‐aged and older people after hypertension and heart disease. Research on anti‐thrombotic food ingredients and thrombolytic drugs is increasing (Xu et al. 2021). Cardiovascular illnesses that are deadly are mostly caused by thrombosis. After cerebral thrombosis, chronic thrombosis can result in cerebral ischemia, hypoxia, softening, and necrosis (Rodríguez et al. 2006). However, the majority of cardiovascular and cerebrovascular disorders develop suddenly and can be quite serious with no prior warning signals. These illnesses, particularly cerebrovascular illnesses, which have a relatively high mortality rate, might result in death if not promptly treated (Oikonomou et al. 2020). In addition, the latest research shows that the adenovirus vector new crown vaccine may induce thrombosis in patients with vaccine‐induced thrombotic thrombocytopenia (VITT). Therefore, thrombosis poses a great threat to human health and warrants greater attention (McGonagle et al. 2021). The preventive and interventional effects of functional foods have become an important research topic. Intraperitoneal injection of carrageenan can cause intestinal inflammation in experimental animals. Inflammation causes the release of several inflammatory mediators and free radicals into the blood, which can harm vascular endothelial cells and result in thrombosis. The mouse tail has a single femoral artery, which makes it difficult for collateral circulation to occur after it is embolized and causes progressive ischemia necrosis of the mouse tail tissues (Arslan et al. 2011). In this work, mice with carrageenan‐induced inflammation developed tail vein thrombosis as a result of the establishment of an animal thrombosis model.

A thrombus may form as a result of inflammation, which will worsen the condition of the inflammation. “Thrombotic inflammation” is the term used to describe this vicious cycle. The creation of reactive oxygen species (ROS) and oxidative stress damage is particularly associated with the onset and development of thrombosis and thrombotic inflammation, which results in the malignant interaction network of oxidative stress‐inflammation‐coagulation (Strukova 2006). The body's sophisticated defensive mechanism against endogenous or foreign damage sources is inflammation. As previously indicated, new research has demonstrated that thrombosis may trigger inflammation, which can then spread even further. The extent of organ damage following thrombosis is influenced by both the original injury and the severity of the ensuing vascular thrombotic inflammation (Tidjane et al. 2015). When thrombotic inflammation is severe, it can travel throughout the body, harm distant organs, and cause multiple organ failure and death. When inflammatory substances are present, the blood becomes hypercoagulable and causes thrombosis (Xia et al. 2010). Additionally, a number of factors involved in thrombosis can contribute to the emergence and progression of inflammation. Inflammation has been identified as a major pathogenic component in a variety of thrombotic illnesses, including acute ischemic stroke, deep vein thrombosis, pulmonary embolism, and cerebral venous thrombosis (DeFilippis et al. 2015; Roumen‐Klappe et al. 2009). Inflammation and oxidative stress are closely linked and promote each other. ROS is a key mediator of inflammation. Inflammation increases ROS generation and produces oxidative stress (Schieber and Chandel 2014). Many thrombotic disorders include oxidative stress in the pathogenic process, including arterial thrombotic diseases such as atherosclerosis, ischemic stroke, and myocardial infarction, as well as venous thrombotic diseases such as deep vein thrombosis and pulmonary embolism (Capra et al. 2014; Madamanchi et al. 2005). ROS‐induced oxidative stress damage to the vascular endothelium is a key element in the initiation and progression of thrombosis. The vascular endothelium produces a substantial quantity of ROS when activated. A high quantity of ROS causes blood vessel damage, increases adhesion molecules, and stimulates and worsens inflammation and thrombosis. Oxidative stress causes thrombosis, which exacerbates oxidative stress (Mezzano et al. 2001).

Unlike traditional anticoagulant drugs that directly inhibit coagulation factors, probiotics such as *Lactobacillus plantarum* exert a mild intervention on thrombosis by regulating the intestinal microecology and reducing inflammatory responses. It is derived from nature and has a multi‐target mechanism of action. Not only can it assist in regulating blood lipids, but it also avoids the bleeding risks that may be caused by drugs, providing a safer and more collaborative solution for the daily management of thrombosis. Lactic acid bacteria have shown potential in the prevention and treatment of inflammation and thrombosis. The core lies in regulating immune pathways and improving vascular function. Specific *Lactiplantibacillus plantarum* and its metabolites can effectively alleviate the inflammatory response in the body by inhibiting the NF‐κB signaling pathway and down‐regulating key pro‐inflammatory factors such as tumor necrosis factor‐α and interleukin‐6 (Liao et al. 2025). This anti‐inflammatory effect is closely related to antioxidant stress. By activating the Nrf2 pathway, it reduces reactive oxygen species and breaks the vicious cycle of “oxidative stress—inflammation—thrombosis” (Liao et al. 2025; Kim et al. 2025). Moreover, some lactic acid bacteria (such as *Limosilactobacillus reuteri* and *Lacticaseibacillus paracasei*) can improve vascular endothelial function. They increase the availability of nitric oxide to maintain vascular dilation and reduce the expression of vascular cell adhesion molecules, thereby inhibiting platelet aggregation and thrombosis (D'Antongiovanni et al. 2026; Jeon et al. 2024).

Sichuan kimchi in China is a naturally fermented vegetable (Cao et al. 2017). According to research, the health advantages of Sichuan kimchi are most likely due to the high lactic acid bacterial gut microbiota it contains. The many varieties of lactic acid bacteria found in Sichuan kimchi are mostly due to Sichuan's unique traits. In addition to the various vegetables used in kimchi production, fermentation temperature, duration, and other parameters, the lactic acid bacteria isolated from Sichuan kimchi differ significantly from other commercially available lactic acid bacteria (Zhang, Yi, et al. 2018; Zhang, Zhou, et al. 2018). Lactic acid bacteria isolated from Sichuan pickles have been shown in studies to have a better colonization effect in the intestinal tract as well as a good preventive and intervening effect on intestinal diseases such as constipation and colitis (Li et al. 2019; Zhang et al. 2019); additionally, these bacteria have a good regulating effect on hyperlipidemia. Lactic acid bacteria are biologically active in the body (Zhu et al. 2019). The drug heparin is often used clinically to treat patients with thrombophilia, so heparin was also selected as the positive drug control in this study. Our team identified a strain of lactic acid bacteria (*Lactiplantibacillus plantarum* CQPC03; LP‐CQPC03) from Sichuan kimchi for this study. LP‐CQPC03 exhibits excellent in vitro resistance, with survival rates exceeding 80% in both simulated gastric acid and bile salts, which are higher than those of most commercially available probiotics, indicating that it is a strain with great probiotic potential (Gan et al. 2020). This study is the first to investigate the antithrombotic effects of LP‐CQPC03. The study observed the microbial gut microbiota in the intestines of mice, as well as the levels of oxidative stress and inflammation. It examined the effect of LP‐CQPC03 on thrombosis, aiming to explore new applications of probiotics in preventing thrombosis, assisting in the elimination of thrombi, and reducing the occurrence of various thrombosis‐related diseases.

## 2 Materials and Methods

### 2.1 Experimental Microorganism Strain

LP‐CQPC03 was isolated from the naturally fermented Sichuan kimchi in Chongqing City, China. The strain was finally identified and deposited at the China General Microbiological Culture Collection Center (Beijing, China); the strain collection number is CGMCC 14492. After the experiment began, LP‐CQPC03 was resuscitated for use.

### 2.2 Animals and Treatments

Fifty Institute of Cancer Research (ICR) 6‐week‐old male mice (23 ± 2 g) were acquired from Chongqing Medical University's Experimental Animal Center (Chongqing, China). The environmental parameters were 20°C ± 1°C, 30%–40% humidity, free access to food and water, a 12 h light/dark cycle, and adaptive breeding for 7 days following the tests. The 50 ICR mice were separated into five groups of 10 mice each: normal, model, heparin (drug positive control), low‐concentration LP‐CQPC03 (LP‐CQPC03‐L), and high‐concentration LP‐CQPC03 (LP‐CQPC03‐H). The mice in the normal group received 0.01 mL/g of physiological saline solution intraperitoneally every day. Every morning at 9:00 for a total of 10 consecutive days, all the experimental mice in the other groups were intraperitoneally injected with 0.01 mL of 0.2% carrageenan solution per gram of body weight (injection volume of carrageenan: 0.01 mL/g b.w.). At the same time, mice in the heparin group received 20 mg/kg of heparin daily, whereas mice in the LP‐CQPC03‐L and LP‐CQPC03‐H groups received 10^8^ and 10^9^ CFU/kg of LP‐CQPC03 daily, respectively. For 10 days, heparin and LP‐CQPC03 were administered through gavage. The length of black in the tail vein (thrombus) in each group of mice was measured after 10 d (Han et al. 2021). Then, using the entering the chamber, the mice were placed in a drying dish and high concentrations of carbon dioxide were introduced to induce suffocation and death. Blood and tissue were then collected for subsequent experiments.

### 2.3 Determination of Blood Coagulation

After the mice were sacrificed, the abdominal skin was cut open at 4°C on an ice bath, and the abdominal cavity was fully exposed. Then, 1 mL of blood was collected from the posterior vena cava using a syringe. Collected plasma was stored in a centrifuge tube containing sodium citrate. The plasma was extracted and the four blood coagulation components activated partial thromboplastin time (APTT), decrease thrombin time (TT), fibrinogen (FIB), and prothrombin time (PT) were identified using a semi‐automated hemagglutination apparatus (PUN‐2048A, Beijing Pulang New Technology Co. Ltd., Beijing, China).

### 2.4 Determination of Oxidation‐Related Indicators

To extract serum, entire blood samples were centrifuged at 4000 rpm for 10 min at 4°C. The oxidation markers were employed in detection kits to determine serum superoxide dismutase (SOD), catalase (CAT), and malondialdehyde (MDA) levels (Shanghai Enzyme Link Biotechnology Co. Ltd., Shanghai, China).

### 2.5 Determination of Inflammation‐Related Indicators

After whole blood samples were processed according to the method described in Section 2.4, the blood were centrifuged at 4000 rpm for 10 min at 4°C, the TNF‐α, IL‐6, NF‐κB, and IL‐1β levels in serum were tested utilizing detection kit (Shanghai Enzyme Link Biotechnology Co. Ltd., Shanghai, China).

### 2.6 Histopathological Observation

Fresh tissue samples were fixed in 4% paraformaldehyde for 48 h. After fixation, they were dehydrated with gradient ethanol, stained with xylene, and embedded in paraffin. Then, they were cut into 4 μm thick sections using a paraffin sectioning machine. The sections were deparaffinized twice with xylene for 10 min each, followed by hydration with gradient ethanol (100%, 95%, 80%) until distilled water. The sections were stained in hematoxylin solution for 5–8 min, rinsed with running water to remove excess color, and differentiated with 1% hydrochloric acid ethanol for a few seconds, then rinsed with running water to blue again for 15 min. Subsequently, the sections were stained in eosin solution for 1–3 min. After staining, they were dehydrated with gradient ethanol, stained with xylene, and sealed with neutral gum. Finally, the tissue was examined under an optical microscope (BX43, Olympus, Tokyo, Japan) to observe the pathological changes (Long et al. 2021). The proportion of inflammatory infiltrating cells in the slices was analyzed using ImageJ 1.44 software.

### 2.7 Determination of mRNA Expression in Mouse Tail Vein Tissue and Colon Tissue Using Quantitative PCR

We properly weighed 0.2 g of tail vein or colon tissues and homogenized them with 9 mL of physiological saline. The RNA was then extracted from mouse tail vein or colon tissues using 0.5 mL of RNAzol (Invitrogen, New York, NY, USA). A superdifferential photometer was used to test the absorbance of the isolated RNA at 260 and 280 nm (Nano‐100, Hangzhou Allsheng Instruments Co. Ltd., Hangzhou, Zhejiang, China). The purity and concentration of RNA were determined, and the concentration of RNA was set to 1 μg/μL. A reaction system comprising 1 μL of cDNA was created after cDNA was generated by reverse transcription. The remaining ingredients were 10 μL of SYBR Green PCR Master Mix, 7 μL of sterile distilled water, and 1 μL of forward and reverse primer solutions (Table 1, Thermo Fisher Scientific, Waltham, MA, USA). A quantitative PCR apparatus (Onestep Plus, Thermo Fisher Scientific) was used to carry out the reaction. 95°C for 60 s; 95°C for 15 s for 40 cycles; 55°C for 30 s; 72°C for 35 s; 95°C for 30 s; and 55°C for 35 s were the reaction conditions. The internal reference was chosen to GAPDH, and its associated genes were examined using the 2^−ΔΔCt^ technique (Hu et al. 2021).

**TABLE 1:** Primer sequence in this experiment.

Gene	Forward sequence	Reverse sequence
Cu/Zn‐SOD	5′‐AACCAGTTGTGTTGTGAGGAC‐3′	5′‐CCACCATGTTTCTTAGAGTGAGG‐3′
Mn‐SOD	5′‐CAGACCTGCCTTACGACTATGG‐3′	5′‐CTCGGTGGCGTTGAGATTGTT‐3′
CAT	5′‐GGAGGCGGGAACCCAATAG‐3′	5′‐GTGTGCCATCTCGTCAGTGAA‐3′
NF‐κB p65	5′‐GAGGCACGAGGCTCCTTTTCT‐3′	5′‐GTAGCTGCATGGAGACTCGAACA‐3′
ICAM‐1	5′‐TCCGCTACCATCACCGTGTAT‐3′	5′‐TAGCCAGCACCGTGAATGTG‐3′
VCAM‐1	5′‐TTGGGAGCCTCAACGGTACT‐3′	5′‐GCAATCGTTTTGTATTCAGGGGA‐3′
E‐selectin	5′‐ATAACGAGACGCCATCATGC‐3′	5′‐TGTCCACTGCCCTTGTGC‐3′
IL‐6	5′‐ATGAAGTTCCTCTCTGCAA‐3′	5′‐AGTGGTATCCTCTGTGAAG‐3′
TNF‐α	5′‐ATGGGGGGCTTCCAGAA‐3	5′‐CCTTTGGGGACCGATCA‐3′
IFN‐γ	5′‐GCTTTGCAGCTCTTCCTCAT‐3′	5′‐GTCACCATCCTTTTGCCAGT‐3′
GAPDH	5′‐TGACCTCAACTACATGGTCTACA‐3′	5′‐CTTCCCATTCTCGGCCTTG‐3′

### 2.8 High‐Throughput 16S rRNA Sequencing of Mouse Intestinal Contents

Fecal samples from each group of mice were collected in sterile centrifuge tubes and stored at −80°C for subsequent microbial and metabolite analysis. Bacterial genomic DNA was extracted from the fecal samples using a DNA extraction kit. The PCR amplification primers were universal primers targeting the V3–V4 region of the 16S rRNA gene (338F/806R). The bacterial primer set included the forward primer 338F (5′‐ACTCCTACGGGAGGCAGCAG‐3′) and the reverse primer 806R (5′‐GGACTACHVGGGTWTCTAAT‐3′). The PCR reaction system consisted of 4 μL of 5× FastPfu Buffer, 2 μL of 2.5 mM dNTPs, 0.8 μL of forward primer (5 μM), 0.8 μL of reverse primer (5 μM), 4 μL of FastPfu polymerase, 0.2 μL of BSA, 10 μg of template DNA, and ddH_2_O added to a final volume of 20 μL. The PCR amplification conditions were as follows: initial denaturation at 98°C for 2 min, denaturation at 98°C for 15 s, annealing at 55°C for 30 s, extension at 72°C for 30 cycles, and a final extension at 72°C for 5 min. The PCR products were recovered using 2% agarose gel electrophoresis and purified with the AxyPrep DNA Gel extraction kit. The purified products were examined by 2% agarose gel electrophoresis and quantified using a Quantus fluorometer. Libraries were constructed from the purified PCR products using the NEXTFLEX Rapid DNA‐Seq kit. Sequencing was performed on the Illumina PE300 platform (San Diego, CA, USA). After quality control and assembly, an average of 50,000 high‐quality sequences were obtained per sample. The Good's coverage index (calculated by Mothur) ranged from 0.97 to 0.99 across all samples, indicating that the sequencing depth was sufficient to capture the majority of bacterial species in the mouse intestinal contents. Raw sequencing reads were quality‐controlled using fastp software and assembled with Flash software. The optimized sequences after quality control and assembly were denoised using the DADA2 plugin in the QIIME 2 pipeline. The sequences obtained after DADA2 denoising were designated as amplicon sequence variants (ASVs). Taxonomic analysis of ASVs was performed using the Naive Bayes classifier in QIIME 2 based on the Silva 16S rRNA gene database (v138), yielding taxonomic annotations. Alpha diversity was analyzed using Mothur (version 1.30), while beta diversity was assessed via principal component analysis (PCA) using the vegan package in R. Species community diversity was analyzed using Python 2.7 (Python Software Foundation, San Jose, CA, USA).

### 2.9 Statistical Analysis

Every experiment was run in three parallel determinations. The average value of the measurement findings was determined, and the standard deviation was calculated. The experimental data were presented as mean ± standard deviation (SD). To identify significant differences among groups, a one‐way analysis of variance (ANOVA) was performed, followed by Tukey's honest significant difference (HSD) post hoc test for multiple comparisons. All statistical analyzes were conducted using SPSS software (version 26.0, IBM, Armonk, NY, USA). A *p*‐value < 0.05 was considered statistically significant.

## 3 Results

### 3.1 Length of Mouse Tail Thromboses

**FIGURE 1:**
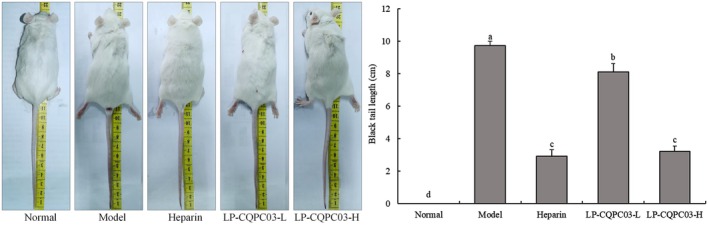
Thrombotic mouse tail thrombus. a–d: According to Duncan's multiple range test, mean values with different letters in the various bars are substantially different (*p* < 0.05).

Following an intraperitoneal injection of carrageenan, a dark colored patch developed at the tip of each mouse's tail (Figure 1), showing tail thrombosis. The mice in the control group had normal tails with no black spots. The black tail was the longest (9.7 ± 0.3 cm) among mice in the model group and substantially longer than the other groups (*p* < 0.05). The black tail length of mice in the LP‐CQPC03‐H group (3.3 ± 0.3 cm) was comparable to that of the heparin group (3.1 ± 0.4 cm), with no significant difference. The LP‐CQPC03‐H and heparin groups had considerably shorter black tail lengths than the LP‐CQPC03‐L group (8.2 ± 0.5 cm, *p* < 0.05).

### 3.2 APTT, TT, FIB, and PT in Mice

**FIGURE 2:**
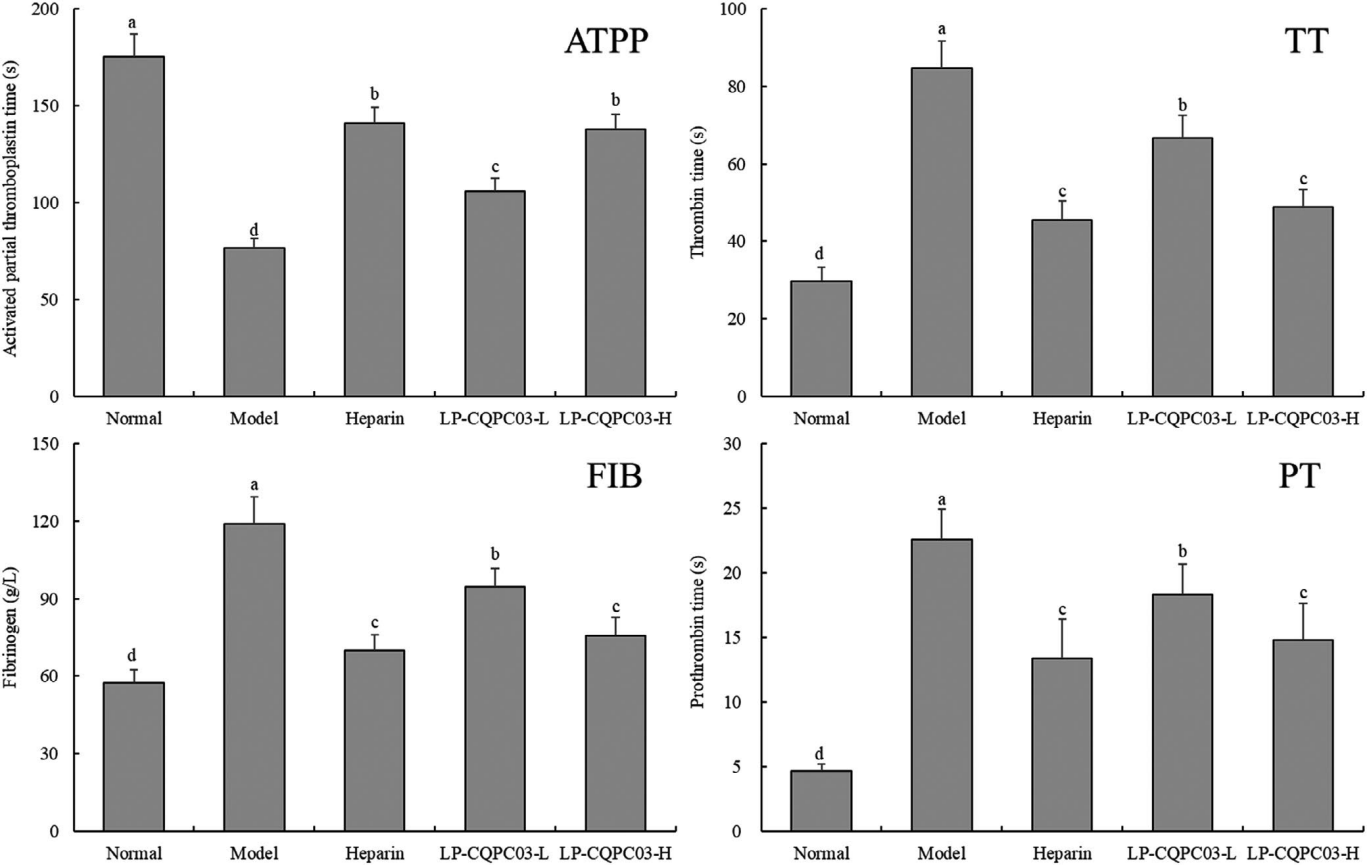
The thrombin time (TT), fibrinogen (FIB), activated partial thromboplastin time (APTT), and prothrombin time (PT) of thrombotic mice. a–d: According to Duncan's multiple range test, mean values with different letters in the various bars are substantially different (*p* < 0.05).

While TT, FIB, and PT were considerably (*p* < 0.05) lower in the normal group of mice than in the other groups, the APTT was significantly (*p* < 0.05) greater (Figure 2) than in the other groups. While TT, FIB, and PT were considerably (*p* < 0.05) greater in the model group than in the other groups, the APTT was significantly (*p* < 0.05) lower in the model group. The APTT of the LP‐CQPC03‐H group was noticeably greater than that of the LP‐CQPC03‐L group, whereas the TT, FIB, and PT were noticeably (*p* < 0.05) lower. There was no discernible difference in the APTT, TT, FIB, and PT in the group receiving LP‐CQPC03‐H and the group receiving heparin (*p* > 0.05).

### 3.3 SOD, CAT, and MDA Levels in Mice

**FIGURE 3:**
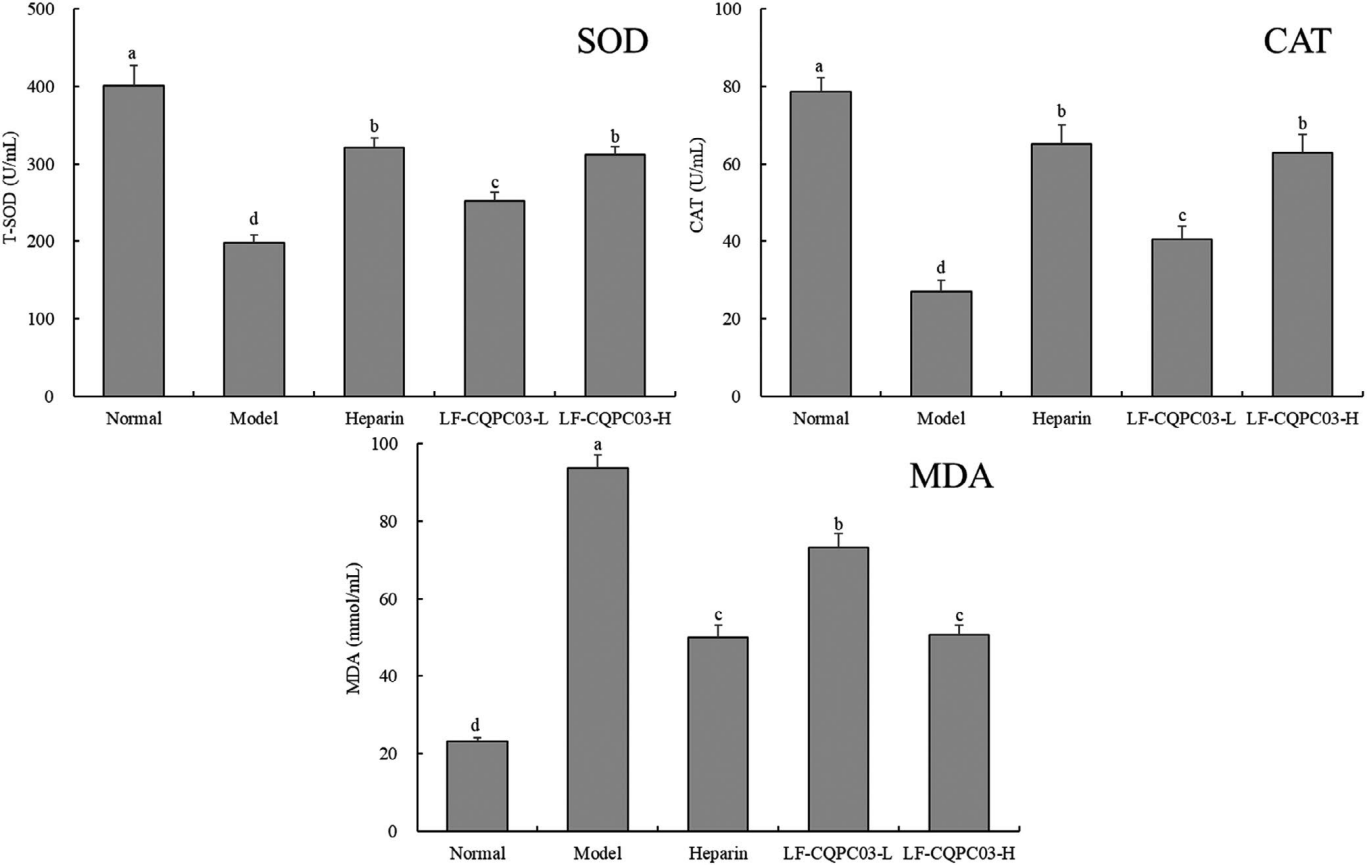
The SOD, CAT, and MDA concentrations in thrombotic mice's serum. a–d: According to Duncan's multiple range test, mean values with different letters in the various bars are substantially different (*p* < 0.05).

The blood test findings revealed that the normal group of mice had the greatest SOD and CAT enzyme activity of all the groups (*p* < 0.05), and the MDA level was the lowest (Figure 3). Heparin and LP‐CQPC03‐H groups had somewhat lower SOD and CAT enzyme activity than the normal group and higher levels than the LP‐CQPC03‐L group (*p* < 0.05). The model group mice had the lowest SOD and CAT enzyme activity (*p* < 0.05). The greatest MDA level was found in the model group, which had a lower MDA level than the LP‐CQPC03‐L group but a higher MDA level than the heparin group and the LP‐CQPC03‐H group (*p* < 0.05).

### 3.4 TNF‐α, IL‐6, NF‐κB, and IL‐1β Levels in Mice

**FIGURE 4:**
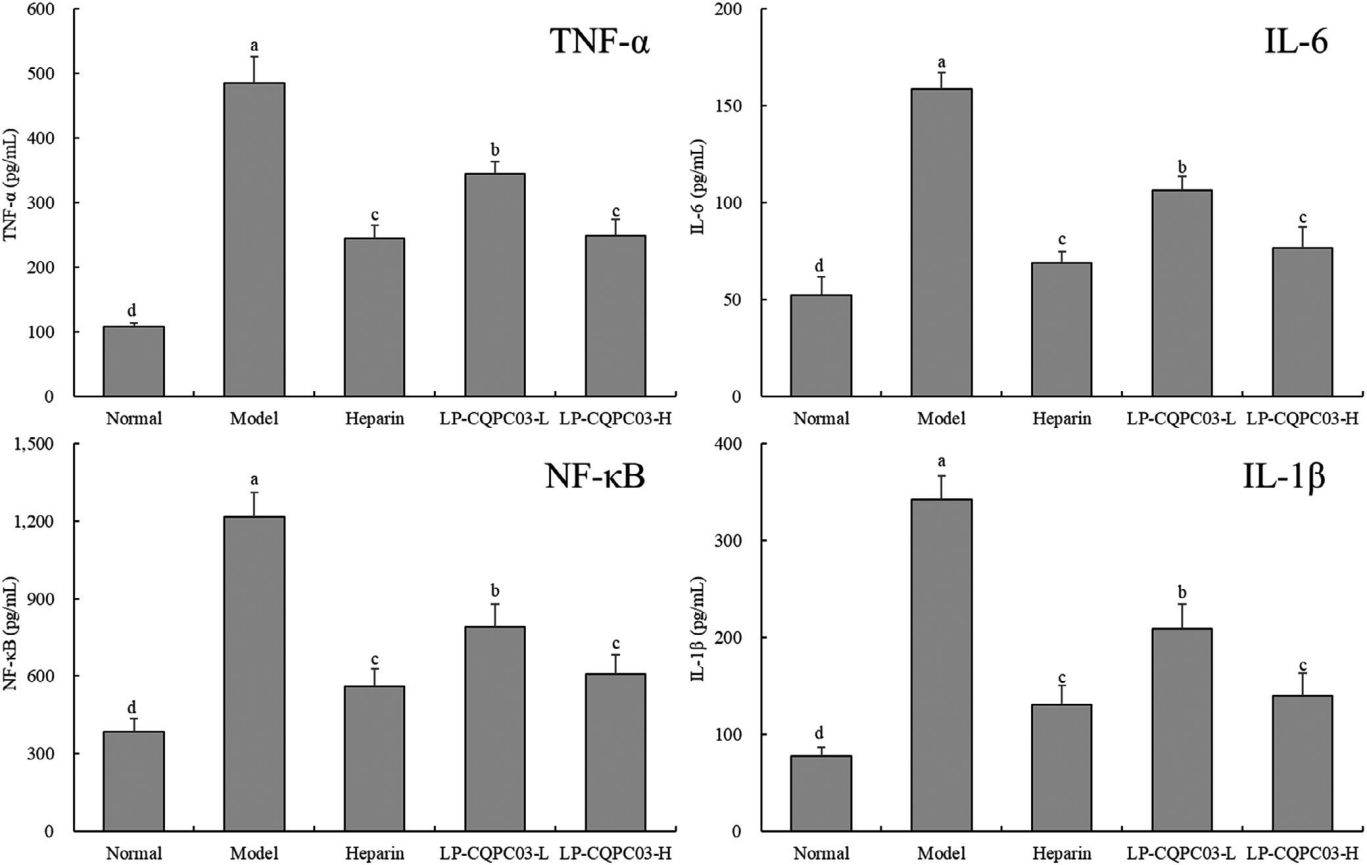
The concentrations of the cytokines TNF‐α, IL‐6, NF‐κB, and IL‐1β in the serum of thrombotic mice. a–d: According to Duncan's multiple range test, mean values with different letters in the various bars are substantially different (*p* < 0.05).

The results showed that the model group showed the highest levels of TNF‐α, IL‐6, NF‐κB, and IL‐1β, while the normal group showed the lowest levels (Figure 4). Compared with the model group, the levels of TNF‐α, IL‐6, NF‐κB, and IL‐1β were reduced by heparin and LP‐CQPC03 (*p* < 0.05), and there was no significant difference between heparin and LP‐CQPC03‐H groups and they were all lower than LP‐CQPC03‐L groups (*p* > 0.05).

### 3.5 Pathological Examination

**FIGURE 5:**
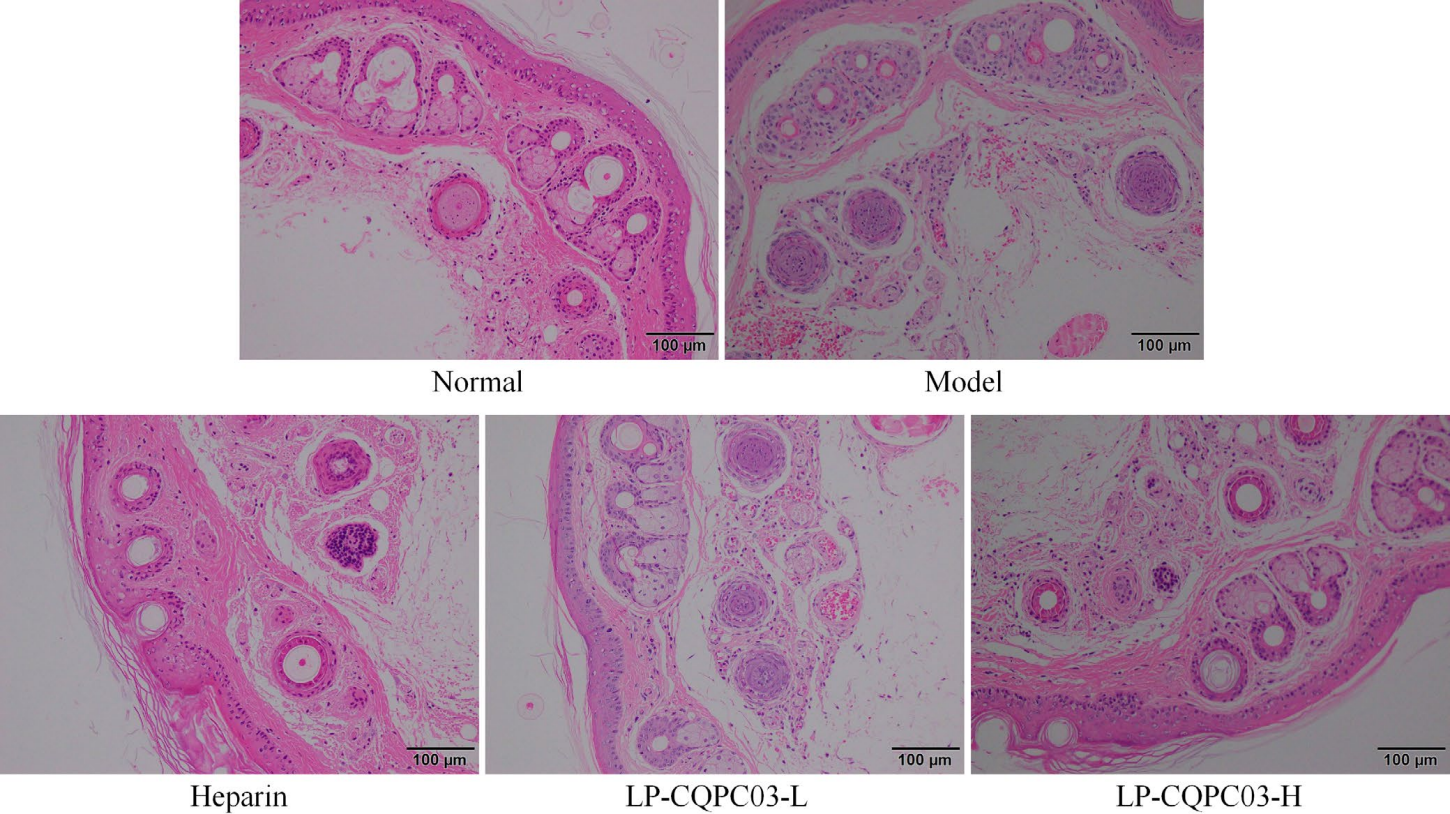
Thrombotic mouse tail vein vessel seen with H&E for pathology.

The tail blood vessels of mice in the normal group were spherical, clean, and the blood vessel walls were smooth on H&E stained sections (Figure 5). White blood cell infiltration, inflammatory exudation, bleeding lesions, platelet aggregation, and thrombosis in the blood vessel wall were all seen in the model group. Heparin and LP‐CQPC03 are both capable of lessening the pathological alterations in the mouse tail blood vessels. Heparin and LP‐CQPC03‐H both had similar effects, while LF‐CQPC03‐H's effects were superior to those of heparin and LP‐CQPC03‐L. Through software analysis of the slices, it was found that the proportions of inflammatory infiltrating cells to the total cell count in the normal, model, heparin, LP‐CQPC03‐L, and LP‐CQPC03‐H groups were 2.24% ± 0.56%, 22.37% ± 2.57%, 11.52% ± 1.87%, 16.89% ± 1.36%, and 12.71% ± 1.66%, respectively.

### 3.6 mRNA Expression of Cu/Zn‐SOD, Mn‐SOD, CAT, NF‐κB p65, IL‐6, TNF‐α, and IFN‐γ in Mouse Colon Tissues

**FIGURE 6:**
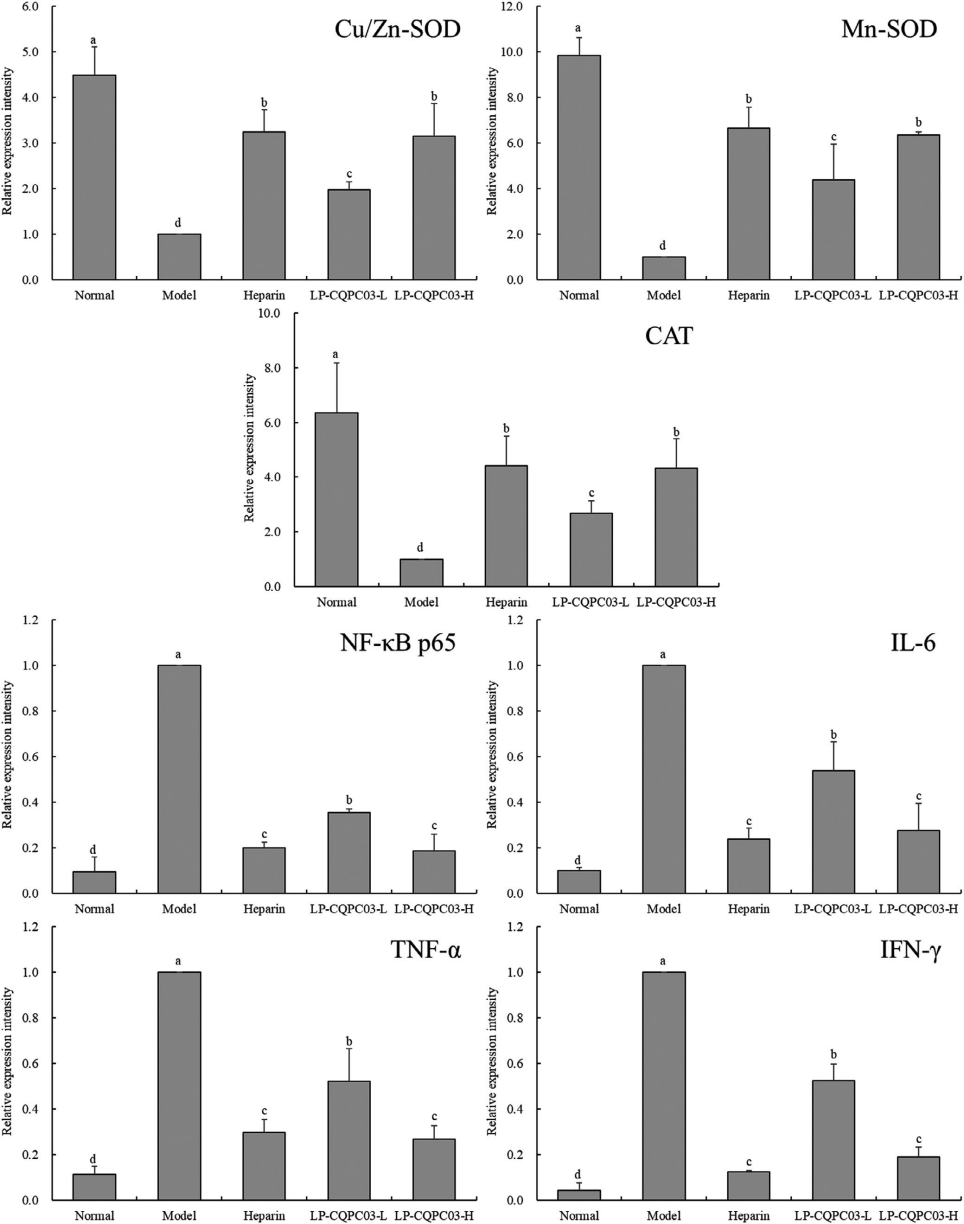
The Cu/Zn‐SOD, Mn‐SOD, CAT, NF‐κB p65, IL‐6, TNF‐α, and IFN‐γ mRNA expression in colon tissues of thrombotic mice. a–d: According to Duncan's multiple range test, mean values with different letters in the various bars are substantially different (*p* < 0.05).

According to the outcomes of qPCR investigations, the Cu/Zn‐SOD, Mn‐SOD, and CAT mRNA expression in the colon tissues of mice in the normal group was the strongest, whereas that in the model group was the smallest (*p* < 0.05, Figure 6). In the colon tissues of mice, Cu/Zn‐SOD, Mn‐SOD, and CAT expression levels were equivalent and stronger (*p* < 0.05) in the LP‐CQPC03‐H and heparin groups than in the LP‐CQPC03‐L group.

The normal group of mice had considerably lower levels of NF‐κB p65, IL‐6, TNF‐α, and IFN‐γ expression in their colon tissues than the other groups, but the model group had significantly greater levels (*p* < 0.05) than the other groups. In the colon tissues of thrombotic mice, LP‐CQPC03 and heparin were both able to suppress the production of NF‐κB p65, IL‐6, TNF‐α, and IFN‐γ. Heparin and LP‐CQPC03‐H both had similar effects, and both were noticeably superior to LP‐CQPC03‐L in terms of effectiveness (*p* < 0.05).

### 3.7 mRNA Expression of NF‐κB p65, ICAM‐1, VCAM‐1, and E‐Selectin in Mouse Tail Vein Tissues

**FIGURE 7:**
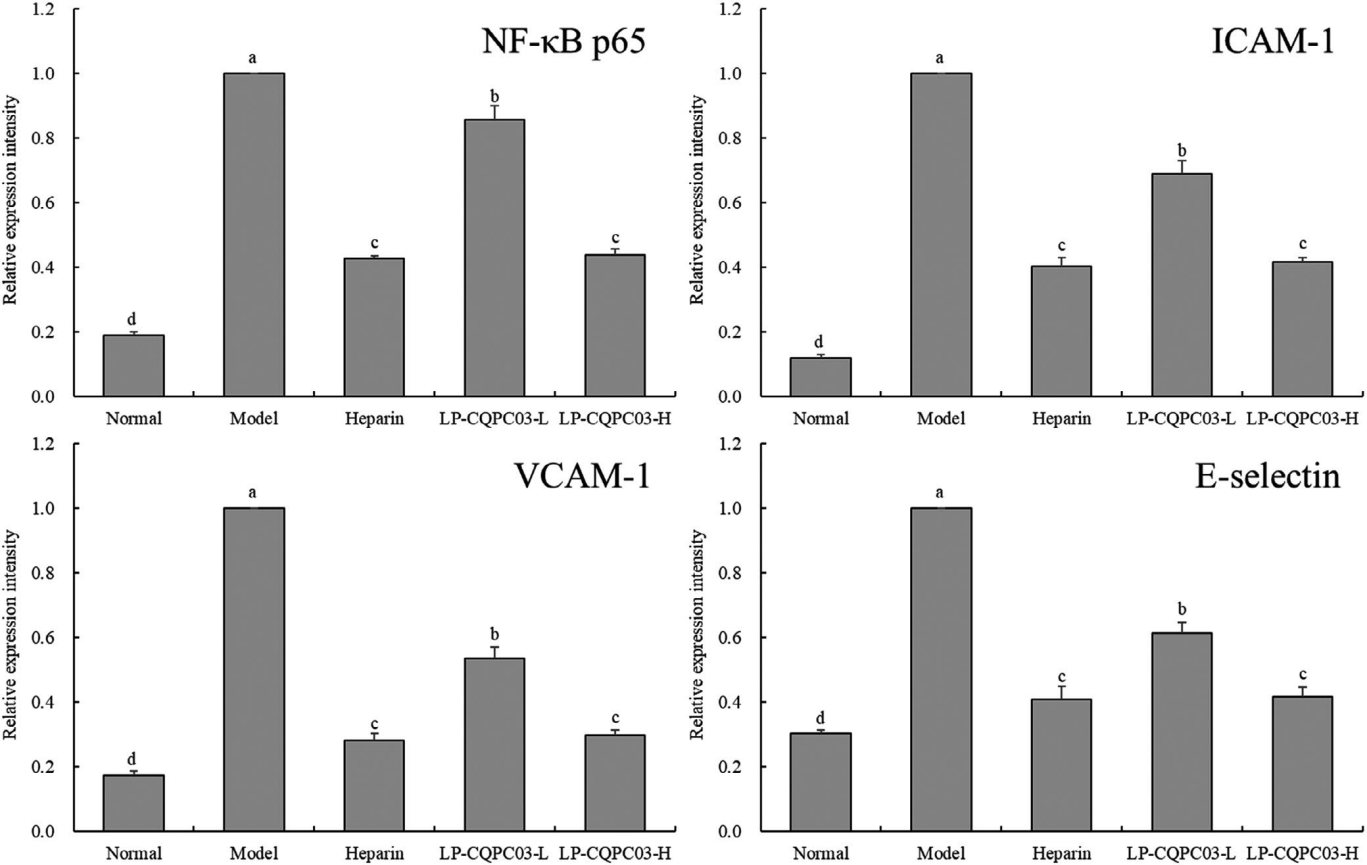
The NF‐κB p65, ICAM‐1, VCAM‐1, and E‐selectin mRNA expression in tail vein tissues of thrombotic mice. a–d: According to Duncan's multiple range test, mean values with different letters in the various bars are substantially different (*p* < 0.05).

According to the results of a qPCR experiment, the model group of mice had the greatest mRNA expression of NF‐B p65, ICAM‐1, VCAM‐1, and E‐selectin in their tail vein arteries (*p* < 0.05, Figure 7). Heparin, LP‐CQPC03‐L, and LP‐CQPC03‐H were able to significantly (*p* < 0.05) reduce the expression of NF‐B p65, ICAM‐1, VCAM‐1, and E‐selectin in the thrombotic mice's tail vein arteries. These expressions were hardly different from those in the normal group and did not differ substantially between animals in the LP‐CQPC03‐H and heparin groups (*p* > 0.05).

### 3.8 Community Diversity Analysis

We obtained information such as species richness and diversity in the samples by evaluating a series of alpha diversity indices. The alpha diversity index for the ACE value, Chao value, Shannon index, and Simpson value showed significant variations across the groups in Table 2 (*p* < 0.05). Both the normal group and the treatment group's digestive contents had more germs than the model group did, according to the research. The concentration of microorganisms varied significantly between the LP‐CQPC03 groups at various dosages. Except for the normal group, the highest bacterial diversity index was that in the LP‐CQPC03‐H group (ACE value: 586.22, Chao value: 581.00). Gut microbiota variety in mice following oral treatment of LP‐CQPC03 was substantially larger than that in the model group, according to an examination of alpha diversity. The LP‐CQPC03‐H group has a Shannon value of 4.16 and a Simpson value of 0.04 according to the data. This showed that the LP‐CQPC03‐H group had much more fecal gut microbiota than the normal group and the heparin group did (*p* < 0.05).

**TABLE 2:** Alpha diversity indices of intestinal microorganisms in the feces of mice.

Group	Sobs	ACE	Chao	Shannon	Simpson	Coverage
Normal	619.00a	670.83a	662.13a	3.86c	0.05b	0.9992a
Model	152.00e	156.69e	156.58e	1.63d	0.37a	0.9999a
Heparin	506.00c	509.62c	507.77c	4.02b	0.06b	0.9999
LP‐CQPC03‐L	384.00d	395.07d	391.32d	3.81c	0.04b	0.9999a
LP‐CQPC03‐H	560.00b	586.22b	581.00b	4.16a	0.04b	0.9996a

*Note:* ^a–e^According to Duncan's multiple range test, mean values with different letters in various columns are substantially different (*p* < 0.05).

**FIGURE 8:**
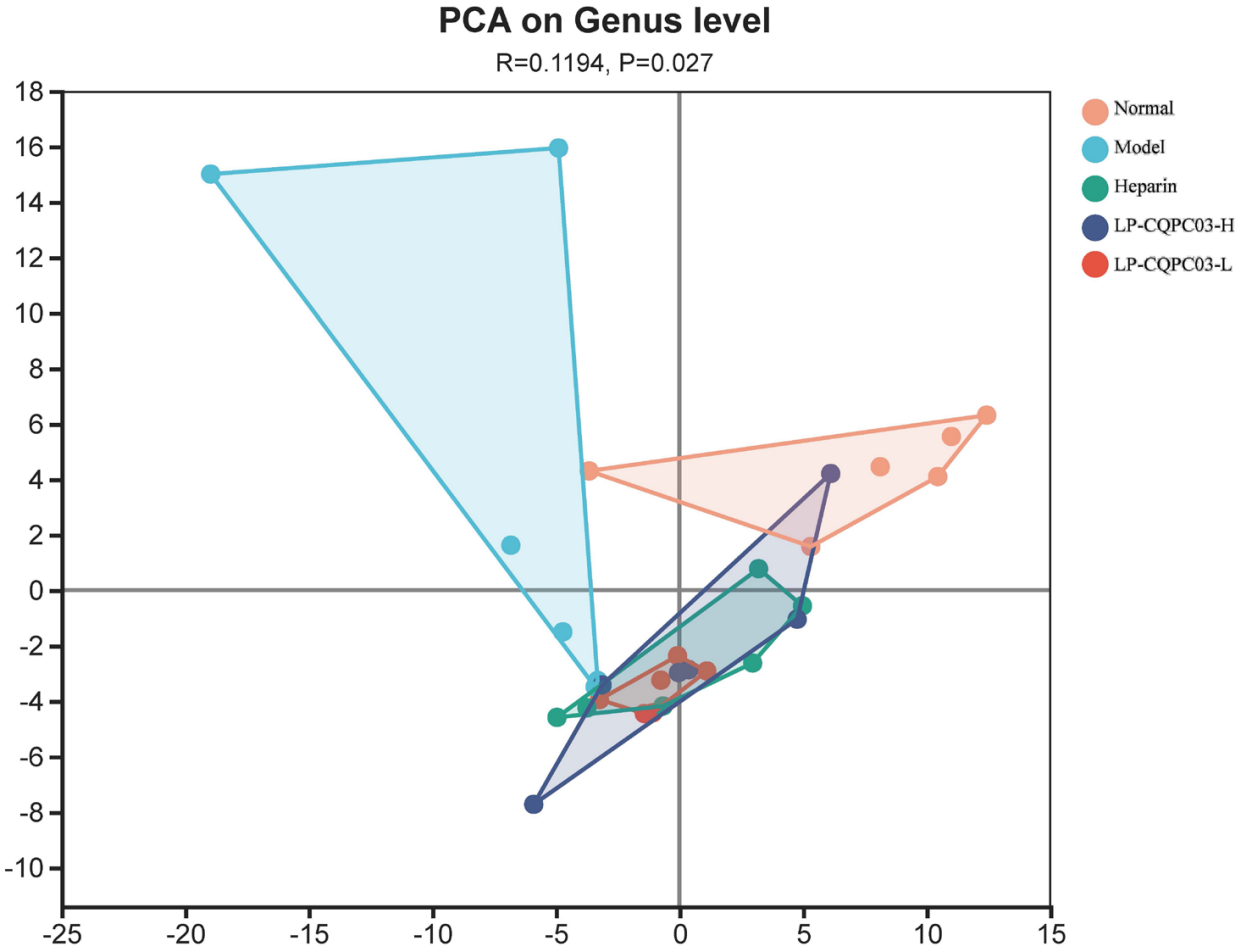
Beta diversity indices of intestinal microorganisms in the feces of mice.

Beta diversity analysis of mouse fecal microbiota revealed that following LP‐CQPC03 intervention, the gut microbial community of mice began to cluster with those of the positive control group and the normal group, while remaining relatively distinct from the model group, with significant differences among the groups (*p* < 0.05, Figure 8). This suggests that LP‐CQPC03 intervention effectively alleviated the gut microbiota dysbiosis in mice.

### 3.9 Community Composition Analysis

**FIGURE 9:**
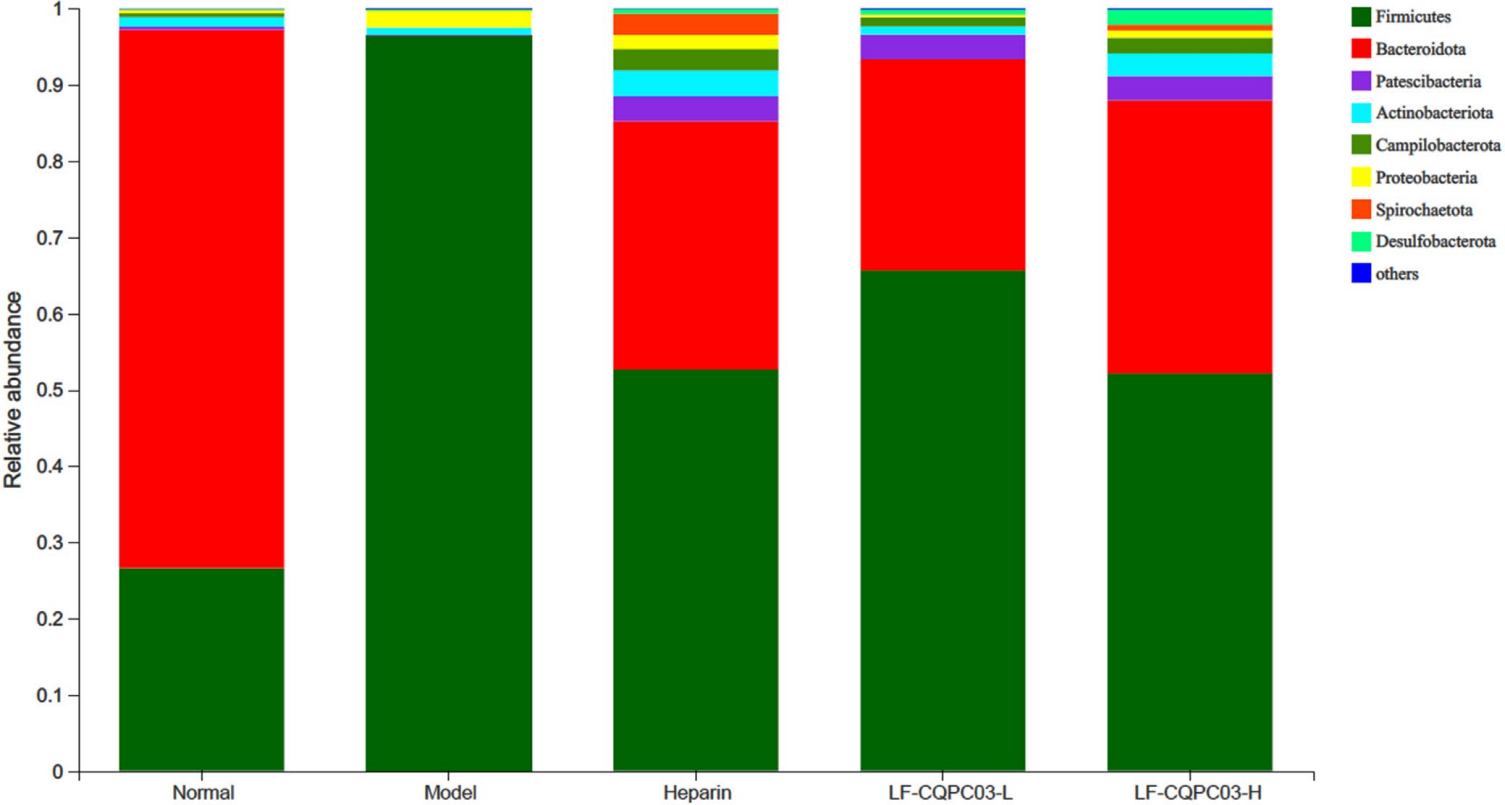
Based on 16S data, the typical bacterial makeup of microbiomes (phylum level).

The community structure composition of various groupings at each taxonomic level was analyzed in light of the findings of taxonomic analysis. As shown in Figure 9, the gut microbiota of the five groups was mainly composed of three phyla: *Bacteroidetes*, *Firmicutes*, and *Actinobacteria*. The lowest *Firmicutes* to *Bacteroidetes* ratio (0.38) was found in the normal group, followed by groups treated with LP‐CQPC03‐H (1.44), LP‐CQPC03‐L (2.36), and heparin (1.61). The outcomes showed that when the dosage of LP‐CQPC03 was increased, the ratio of *Firmicutes* to *Bacteroidetes* dropped (*p* < 0.05).

**FIGURE 10:**
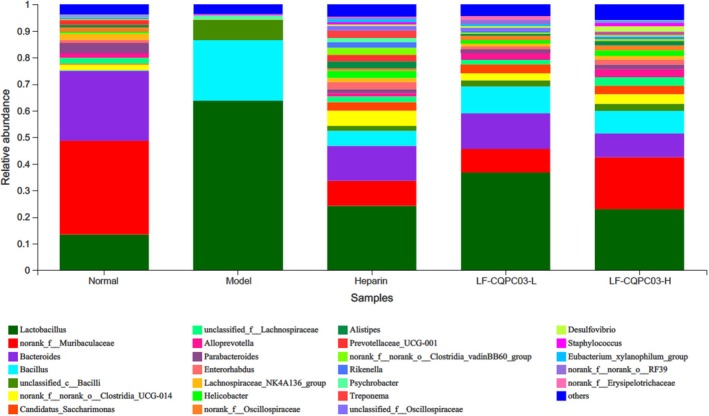
Based on 16S data, the typical bacterial makeup of microbiomes (genus level).

The composition of gut microbiota at the genus level in the five groups is shown in Figure 10. In the mouse thrombosis model induced by carrageenan, there were significant differences in the characteristics of the intestinal microbiota among the various groups: the normal group had a balanced microbiota structure, mainly consisting of beneficial bacteria such as *norank_f_Muribaculaceae* and *Bacteroides*, with a moderate abundance of *Lactobacillus*; the model group showed a significant microecological imbalance, with abnormal increases in the abundance of *Lactobacillus* and *Bacillus*, and a sharp decline in microbiota diversity. This is closely related to the inflammatory response, oxidative stress, and intestinal barrier damage triggered by the thrombus, and is a compensatory proliferation under the disease state. The gut microbiota in the LP‐CQPC03‐H group included *Bacteroides*, *Lactobacillus*, *Alistipes*, and unclassified *Lachnospiraceae*. After oral administration of LP‐CQPC03, *Lactobacillus* in the intestinal contents of mice increased significantly (*p* < 0.05). The most pronounced rising effect was seen in the LP‐CQPC03‐H group, which was comparable to the normal group. Additionally, when LP‐CQPC03 was administered orally, less pathogenic bacteria, including *Klebsiella*, were present compared to the model group (*p* < 0.05). This tendency continued as LP‐CQPC03 dosage was raised. The heparin group and the *Lactiplantibacillus plantarum* LP‐CQPC03 intervention group (especially the high‐dose group) can significantly reverse the gut microbiota imbalance caused by carrageenan, promote the proliferation of beneficial bacteria such as *Alloprevotella* and *Parabacteroides*, and restore the diversity of the gut microbiota.

## 4 Discussion

The mouse thrombosis model induced by carrageenan is a classic animal model widely used in the research of antithrombotic drugs. Its core mechanism lies in that carrageenan, as a specific ligand, can directly bind to the C‐type lectin‐like receptor 2 (CLEC‐2) on the surface of platelets, activating the tyrosine kinase‐dependent pathway (Syk, LAT, PLCγ2) and leading to platelet aggregation and thrombus formation in tail vessels. Yokomori et al. (2023) demonstrated using surface plasmon resonance that κ‐carrageenan directly binds to recombinant CLEC‐2 protein, and CLEC‐2‐deficient mice show significantly reduced thrombosis and thrombocytopenia. Due to vessel obstruction, tail tissue exhibits purplish‐black necrosis, and researchers quantify thrombus degree by measuring “black tail length”. The advantages of this model include clear mechanism, simple operation (intraperitoneal injection), intuitive indicators, and multi‐dimensional evaluation (histopathology, molecular detection). Additionally, this model accompanies significant inflammatory response, suitable for studying thrombus‐inflammation interaction. Both heparin and LP‐CQPC03 lessened thrombosis‐related black tail, with high‐concentration LP‐CQPC03 having a stronger impact, approaching the effect of heparin.

In the mouse model of carrageenan‐induced thrombosis, the model group typically exhibits a significantly shortened activated partial thromboplastin time (APTT) and a significantly increased fibrinogen (FIB) level, accompanied by significantly prolonged thrombin time (TT) and prothrombin time (PT) (Jing 2020). When the hypercoagulable state is alleviated, the changes in these parameters reflect the mechanism by which the coagulation system shifts from imbalance to equilibrium. Specifically, APTT, as a sensitive indicator of the intrinsic coagulation pathway, is shortened in the model group, suggesting enhanced activity of factors VIII, IX, XI, and XII or a hypercoagulable state. The prolongation of APTT following improvement indicates effective inhibition of the overactivation of intrinsic coagulation factors. PT represents the extrinsic coagulation pathway, and its significant prolongation in the model group may be related to the consumption of coagulation factors; its recovery following improvement suggests effective regulation of the activation of the extrinsic pathway. TT reflects the final common pathway of converting fibrinogen to fibrin, and its prolongation in the model group may indicate abnormal fibrin formation; its shortening after intervention suggests that the activity at the end of the coagulation cascade is returning to normal. FIB, as a key substrate in the coagulation process and an acute‐phase reactant, is significantly elevated in the model group. A decrease in FIB following improvement directly implies a reduction in the coagulation substrate and the alleviation of inflammatory stress. Therefore, the synergistic improvement of these four parameters—prolongation of APTT, PT, and TT, along with a decrease in FIB—collectively reveals the coagulation mechanisms underlying the remission of the hypercoagulable state in the carrageenan‐induced thrombosis model (Wang et al. 2009, 2016). Heparin and LP‐CQPC03 may be able to prevent thrombosis since they were involved in regulating the four blood coagulation indicators in this investigation. The impact of LP‐CQPC03 also rises with concentration.

Accumulation of free radicals has a significant role in both causing and escalating thrombosis. Platelets are substantially more sensitive to platelet aggregation agents such thrombin, collagen, and arachidonic acid when exposed to ROS, in addition to directly activating platelets (Yamashita et al. 2015). In addition, the ROS produced by white blood cells can encourage thrombosis by causing platelet and white blood cell aggregation. Additionally, ROS can cause venous thrombosis, induce endothelial cell death, stimulate the NF‐B signaling pathway, and enhance the release of thrombus molecules (Xia et al. 2014). Free radical scavengers may also entirely prevent thrombosis brought on by iron ions, demonstrating the critical role that oxidative stress plays in thrombosis. SOD is essential for maintaining a healthy balance between oxidation and anti‐oxidation in the body because it can catalyze the disproportionation of superoxide anion radicals to produce oxygen and hydrogen peroxide. Mammals produce two significant SOD types: Cu/Zn‐SOD and Mn‐SOD (Wolin et al. 2005). The enzymatic activity of CAT, an enzyme scavenger that may encourage the breakdown of H_2_O_2_ into molecular oxygen and water, gives the body an antioxidant defense (Ledesma et al. 2012). SOD and CAT are vital antioxidant enzymes that protect the body from ROS‐induced harm and are also useful active ingredients that encourage blood clots brought on by oxidative stress (Olsvik et al. 2005). Free radicals interact with lipids in living things to produce peroxidation. MDA, the last byproduct of oxidation, causes proteins, nucleic acids, and other organic macromolecules to cross‐link and polymerize. Because MDA is cytotoxic, its concentration can both directly and indirectly indicate how much lipid peroxidation has occurred in the body. The MDA level can possibly be a crucial thrombosis indication (Chen et al. 1995). In this study, mice with induced thrombosis exhibited higher body levels of oxidative stress, lower levels of SOD and CAT antioxidant enzymes and mRNA expression, and higher levels of MDA, which were similar to those in other studies. These findings imply that thrombosis and oxidative stress are tightly connected. At high concentrations, LP‐CQPC03 could help these oxidation‐related indicators reach levels close to the normal state as its effect reached the level of antithrombotic drugs.

Inflammation can lead to the creation of thrombus, and thrombus will worsen the progression of inflammation. “Thrombotic inflammation” is the term used to describe this vicious cycle. TLR may activate monocytes, macrophages, lymphocytes, and endothelial cells to enhance the generation of TNF‐α, IL‐6, and other inflammatory mediators after inflammation has already taken place (Franks et al. 2013). Among these, TNF‐ is a crucial mediator linking thrombus and inflammation in addition to having a significant impact on the body's immune‐inflammatory coordination signal network. First, TNF‐α regulates the downstream NF‐κB and MAPK signaling pathways through its receptor as the catalyst of the inflammatory cell cascade. This promotes macrophage release of IL‐6, IL‐8, IFN‐γ, and other inflammatory factors and induces the activation of various cells, including macrophages and lymphocytes, which worsens vascular endothelial damage and deep vein thrombosis (Sochorová et al. 2007). TNF‐α also encourages endothelial cells to generate IL‐1 and IFN, which damages vascular endothelial cells and causes platelet adhesion and thrombosis. In addition to decreasing the transcription and expression of TM in endothelial cells and weakening its anticoagulant function, cytokines like TNF‐α and IL‐1β also drive the production of vasoconstrictor substances, lead to vasoconstriction, and encourage the development of thrombi (Chen et al. 2018). Therefore, controlling the inflammatory response and preventing thrombus development is possible through the modulation of TNF‐α, IL‐6, NF‐B, and IL‐1β. It is also possible to limit thrombus formation. In this study, the drug heparin interfered with the abovementioned inflammatory cytokines, which resulted in an inhibitory effect on thrombosis. Furthermore, the high concentration of LP‐CQPC03 played a similar role, with a good intervention effect on inflammatory cytokines and thrombosis. It can be seen that thrombus formation and development is a complex process involving the interaction of vascular endothelial cells, platelets, white blood cells, and the physical environment. Its mechanism is closely related to the “malignant interaction network of oxidative stress–inflammation–coagulation.” Blocking this network is an important breakthrough for the development of new and effective antithrombotic drugs. As a healthy natural microorganism with no side effects, LP‐CQPC03 may play a similar role in blocking the formation of thrombus.

Carrageenan can cause local inflammation in mice, especially intestinal inflammation, and it can also cause endothelial cell damage. Therefore, observing the degree of intestinal damage is also a means of judging the degree of experimental thrombosis caused by carrageenan (Wei et al. 2006). The observation of this study showed that after the formation of tail thrombosis in mice, the expression of inflammation in the colon tissue of mice changed, which once again proved that tail thrombosis in mice is closely related to intestinal lesions. Both the drug heparin and LP‐CQPC03 could inhibit these pathological changes. Thus, with probiotic potential, LP‐CQPC03 could play a role close to that of heparin.

The inflammatory process in deep vein thrombosis depends heavily on NF‐κB. Inflammatory responses, platelets, and endothelial cells interact through NF‐κB, upsetting the balance between coagulation and fiber melting and causing thrombosis (Gan et al. 2020). ICAM‐1 is a key player in the initiation and progression of inflammation because it primarily facilitates the adhesion response between cells and the matrix (Kevil et al. 2004). VCAM‐1 can cause inflammatory responses at thrombus locations and adversely influence platelet adhesion and aggregation (ten Hacken et al. 1998). In the presence of blood flow, E‐selectin can mediate the local adhesion of leukocytes and vascular endothelial cells, cause inflammatory damage to endothelial cells, and enhance their permeability, and hasten leukocyte exudation (Roth Flach et al. 2013). The NF‐κB signaling pathway is the key component of several inflammatory reactions. The NF‐κB pathway can activate endothelial cells to activate the inflammatory response, which increases the expression of adhesion molecules and cytokines like ICAM‐1, VCAM‐1, and E‐selectin and further activates NF‐κB to amplify the inflammatory response, activate platelet aggregation and coagulation reaction, and form a hypercoagulable state (Xia et al. 2001). This pathway can also up‐regulate the expression of proinflammatory factors in the activated state. In this study, inflammation in the mice's tail vein was brought on by thrombosis. The associated expressions of ICAM‐1, VCAM‐1, and E‐selectin in the NF‐κB‐centric pathway were shown to be substantially different from the baseline condition in this investigation. Heparin and LP‐CQPC03 both have the potential to control the expression of NF‐κB, ICAM‐1, VCAM‐1, and E‐selectin, potentially having beneficial thrombosis inhibitory effects.

The gut microbiota are involved in immune function, detoxification, inflammation, and neurotransmission. Gut microbiota are closely related to inflammation and antioxidant capacity, and their imbalance is linked to cardiovascular risk factors such as hyperlipidemia, obesity, and type 2 diabetes (Wang et al. 2011). Our study also showed that a thrombus led to changes in the abundance of gut microbiota in mice, and LP‐CQPC03 could regulate the bacterial abundance in mice with thrombosis and restore intestinal health, which may play a role in inhibiting thrombus formation. Recent research has elucidated molecular mechanisms linking gut microbiota to thrombosis; they reported that intermittent fasting increases indole‐3‐propionic acid (IPA) from *Clostridium sporogenes* , which binds to platelet pregnane X receptor (PXR) to inhibit platelet aggregation. Another study showed that decreased deoxycholic acid (DCA) from *Bacteroides vulgatus* reduces platelet activation via the TGR5 receptor (Qi et al. 2025). Additionally, Huang et al. (2025) found that high‐fat diet induces *Bacteroides thetaiotaomicron* to produce palmitic acid, which inhibits activated protein C (APC) and promotes thrombosis. These findings provide new molecular targets for comparing the effects of LP‐CQPC03 in the future.

*Parabacteroides* are related to promoting obesity (Wang et al. 2019), and *Klebsiella* are the second most important conditional pathogens after *Escherichia coli* (Shanthi and Sekar 2010). The experimental results showed that there were more harmful bacteria in the intestinal tract of model mice. *Lactobacillus* can be used as a probiotic (Rezazadeh et al. 2021), and the intestinal tract of normal mice contained many beneficial *Lactobacillus*. *Alistipes* are proven to have the effect of interfering with inflammation (Wang et al. 2020), whereas *Lachnospiraceae* have shown a protective effect on the hematopoietic system and intestinal system (Kouidhim et al. 2021). These are important beneficial bacteria in the human body. LP‐CQPC03 increased the number of beneficial bacteria in the intestine of thrombotic mice by increasing *Lactobacillus*, *Alistipes*, and *Lachnospiraceae* in the intestine of these mice, which might enhance immunity and reduce inflammation so as to inhibit thrombosis.

The abundance of normal mouse *Lactobacillus* was lower than that of the model group. In a healthy intestine, Lactobacillus maintains a moderate level and works in synergy with other microorganisms. However, in the thrombosis model, inflammation and oxidative stress drive abnormal proliferation of Lactobacillus as an adaptive response (Li et al. 2025; Paszti‐Gere et al. 2012). Probiotics can reduce the total abundance of lactobacilli and reshape the microbial community through niche competition, immune regulation, and microbial interactions, thereby inhibiting platelet activation and thrombosis via the gut‐vascular axis (Alexandrescu et al. 2024; Zhang et al. 2025). *Lactiplantibacillus plantarum* CQPC03 demonstrated similar effects and mechanisms, providing new directions for intervention in thrombosis‐related diseases. However, a deeper understanding of the mechanism still requires further research.

## 5 Conclusion

In this work, we looked at the recently identified LP‐CQPC03's to prevent mouse thrombosis. This study demonstrated that LP‐CQPC03 can control mouse intestinal function and aid in the development of a better intestinal microbial composition, preserving bodily health by lowering oxidative stress and inflammation and having a strong thrombosis‐inhibitory impact. This is the first report demonstrating that a probiotic *Lactiplantibacillus* strain (LP‐CQPC03) exerts antithrombotic effects through multi‐target regulation of coagulation, oxidative stress, inflammation, and gut microbiota, highlighting its novelty as a natural, safe, and side‐effect‐free alternative to conventional antithrombotic drugs. The findings suggest that LP‐CQPC03 holds significant potential for development as a functional food supplement or probiotic‐based preventive strategy for thrombosis‐related disorders, particularly in individuals with high‐risk factors such as hyperlipidemia or chronic inflammation. However, to validate the results of this investigation, more clinical studies and human trials are required.

## Author Contributions

**Xin Zhao:** writing – original draft, visualization, investigation, formal analysis, conceptualization. **Kejia Wang:** methodology, investigation, formal analysis. **Tianyi Chen:** writing – original draft, investigation. **Kai Ma:** writing – original draft, investigation. **Rongming Zhang:** writing – original draft, investigation. **Yongling Ou:** writing – original draft, investigation. **Zhiping Kuang:** validation, supervision, resources, project administration, funding acquisition.

## Funding

This work was supported by Chongqing Program for Supporting Returned Overseas Students to Start Businesses and Make Innovations (Grant cx2022030) and Chongqing Municipal Education Commission Science and Technology Research Project (Grant KJZD‐M202201601).

## Ethics Statement

All operations were in line with the breeding process of experimental mice, and other experimental procedures were in line with the ethics requirements for the use of experimental animals (approval number: 202107001B).

## Conflicts of Interest

The authors declare no conflicts of interest.

## Data Availability

The data supporting the findings of this study are available from the corresponding author upon reasonable request. 16S rDNA sequencing data of *Lactiplantibacillus plantarum* CQPC03 is saved in the NLM‐NCBI appropriate database (https://www.ncbi.nlm.nih.gov/nuccore/ON359959).
